# The Relationship between Norms and Risky Driving Behavior: A Systematic Review

**Published:** 2020-02

**Authors:** Siti Hawa HARITH, Norashikin MAHMUD

**Affiliations:** 1. Department of Human Resource Development and Psychology, Faculty of Social Sciences and Humanities, Universiti Teknologi Malaysia, Johor, Malaysia; 2. Department of Human Resource, School of Business Management, Universiti Utara Malaysia, Kedah, Malaysia

**Keywords:** Subjective norm, Moral norm, Group norm, Injunctive norm, Descriptive norm

## Abstract

**Background:**

Road accident statistics has been seen increasing over the years despite numerous efforts made by the authorities. Human factors have contributed 90% of accident occurrence with risky driving behavior being one of the significant human factors that can be further explained through norms. This review paper aimed to investigate the relationship between norms and drivers’ risky driving behavior.

**Methods:**

A systematic review process was conducted through four academic databases namely Scopus, Wiley Online Library, Emerald and Web of Science of no limitation for date. Overall, 3443 titles were identified and after several screening and reviewing processes, only 27 studies were included.

**Results:**

The results of the review demonstrated mixed findings between subjective norm and risky driving behavior, whereas the relationship between group norm, moral norm, injunctive norm, descriptive norm and risky driving behavior were observed significant.

**Conclusion:**

Appropriate educational awareness is required to educate the society in practicing good norms for mutual benefit of the society. Parents also need to set a good example for their children by abiding the traffic rules and regulation.

## Introduction

Road accident is a global issue that has become one of the world agenda. WHO further reported that every year, more than 1.25 million people were killed in road accidents; this statistics was not seen reducing for the past years despite numerous efforts taken by the United Nation. Road accidents may cause a major loss not only to the society, but also to the employers and country. Human factors caused 90% of the road accidents, divided into individual and social factors (1, 2). Risky driving behavior is the drivers’ intentional violation of traffic rules and regulation (3, 4).

Drivers’ risky driving behavior can be explained by applying Attribution Theory (AT), which suggests that human behavior occurs due to internal and external attribution (5). Internal attribution involves the factors within the individual themselves, such as personality, traits and abilities, whereas external attribution involves the outside factors that can influence one’s behavior such as the environment and social norm (5). Norms can be categorized as individual and social factors. In general, norm is defined as the informal guidance or standard of doing the rightfulness and avoids the wrongfulness (6, 7).

Subjective norm, group norm, moral norm, injunctive norm and descriptive norm can lead to risky driving behavior. Subjective norm can be explained through the situation when an individual perceives that his/her peer/family want them to commit or avoid such behavior (8). Descriptive norm is an expectation that people will behave in good behavior and avoid bad behaviour, whereas the injunctive norm is a rule that specifies what behavior ought to be done and not to be done (9). These three norms specify the function of significant others to influence an individual to behave accordingly. Similarly, group norm also focuses on the third parties; for example, being in a group of peers or family members who drive aberrantly will likely cause the individual to drive in a similar manner (10).

In contrast, moral norm focuses on the individual himself/herself in committing such behaviour. Moral norm is defined as an individual obligation towards performing the right behavior and avoiding the wrong behavior (6, 7). Drivers with a high moral sense of obligation will abide by the traffic rules and regulations, which consequently would prohibit them from involving in road accident without regard to any other external factors including vehicle technical faulty or environmental factor (11, 12). Norms indeed play a significant role in influencing drivers’ risky driving behavior that could further lead towards the occurrence of road accident. To address this issue, this study proposed to investigate the effects of norms toward drivers’ risky driving behaviour.

We aimed at systematically reviewing the relationship between different types of norms (subjective norm, group norm, moral norm, injunctive norm and descriptive norms) and risky driving behaviour.

## Methods

A systematic review of literature was conducted using four academic databases of Scopus, Wiley Online Library, Emerald and Web of Science.

Several search terms used in this review process were “norms”, “risky driving behaviour” and “violation driving behaviour”. The time span for the search was within the year 1970 up to 2018 and limited to the articles written in English. Following the search strategy process, all retrieved findings were then being exported into the reference management software, EndNote X7. Next, the researchers independently screened each of the titles and abstract of related findings in order to finalize the relevant papers. The inclusion and exclusion criteria were as follows:

*Eligibility criteria:-*
  1. This review only includes cross-sectional studies.
  2. This review includes all types of participants/respondents (drivers) who commit the behavioural outcome of risky driving behaviour.
  3. All types of behavioural outcomes are included in this review.
  4. This review includes all studies that study on subjective norm, group norm, moral norm, injunctive norm and descriptive norm with risky driving behaviour.
*Exclusion criteria:-*
  1. This review excludes meta-analysis paper, systematic review paper, students’ dissertation and governmental report.

## Results and Discussion

### Search Result

**Fig. 1:**
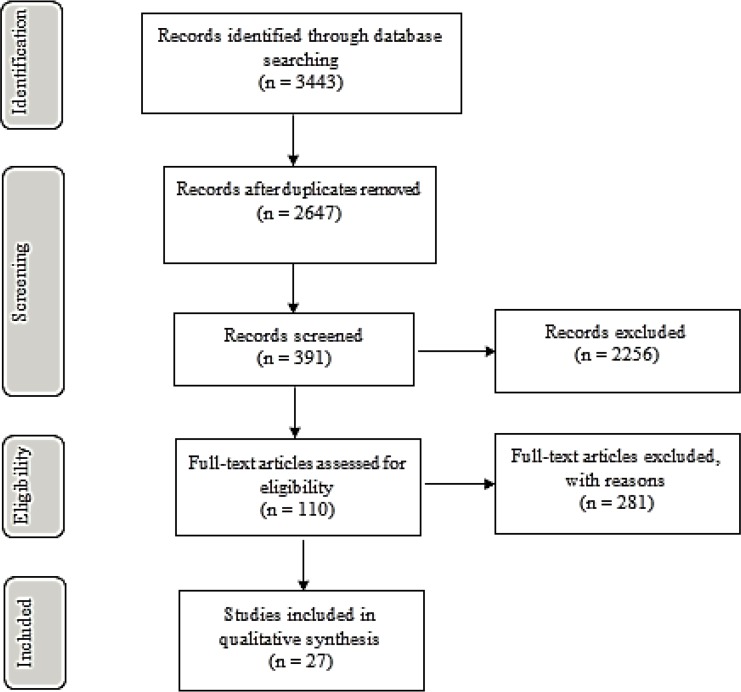
PRISMA flow diagram of the included and excluded studies

Overall, 3443 findings were identified from the literature search process. Totally, 362 findings were identified from Scopus, 462 findings from Wiley Online Library, 746 from Emerald and finally 1873 from Web of Sciences. The time span of all findings was within the year 1970 up to 2018. From the overall findings, this study eliminated 796 duplicate findings during the identification process. Next, the remaining 2647 titles were put into titles screening process wherein this stage, 2256 irrelevant titles were removed. Subsequently, the remaining 391 abstracts were read with 281 irrelevant abstracts that were then re-moved. Then, the full text of the remaining 110 related abstracts was read and all the full texts were filtered to match with the eligibility criteria and exclusion criteria. Finally, only 27 studies were selected for this review paper. Fig. 1 illustrates the PRISMA flow diagram for the included and excluded studies.

### Research setting

All included studies were undertaken in 12 countries worldwide with the majority of the studies (n=7) were conducted in France (8, 13–18), five in the United Kingdom (19–23), three studies in Sweden (24–26), two in Taiwan (27, 28), Australia (10, 12) and Norway (6, 7) and finally one in Malaysia (29), United States (9), Spain (30), Turkey (31), Iran (11) and Uganda (32).

### Participants

All included studies recruited drivers who either had been involved in any road accident or not. Six studies used the sample of adolescence/young drivers (10, 12, 14, 16–18) and general drivers (authors not specifically mention the type of sample) (8, 11, 19, 24, 25, 32), four studies used the sample of motorcyclists (15, 27, 29, 31), three studies used the sample of random residents (6, 7, 9), two studies used the sample of the traffic offender (20, 28) and university/college students (23, 30) and finally one study used the sample of cyclists (13), truck drivers (21), male drivers (22) and test driver participants (26).

### Type of norms and behavioural outcomes

From all 27 included studies, 20 studies investigated the relationship between subjective norm and risky driving behaviour, three studies investigated on the relationship between group norm and risky driving behaviour, seven studies investigated the relationship between moral norm and risky driving behaviour, six studies investigated the relationship between injunctive norm and risky driving behavior and finally, nine studies investigated the relationship between descriptive norm and risky driving behaviour. The behavioural outcomes of risky driving behavior in this review are speeding, dangerous overtake, used of mobile phone while driving, drink-drive, tailgating, disobey road sign, run over red light and neglect the helmet usage.

### Summary on the Relationship between Subjective Norm and Risky Driving Behaviour

Table 1 summarized the outcomes of the relationship between subjective norm and risky driving behaviour. In overall, most studies with significant findings were undertaken in Western and European countries such as Sweden, France, Australia, Norway and UK, whereas both of the insignificant studies were undertaken in Asian countries specifically Taiwan. Thus, research setting also plays a role in influencing the final outcome since different countries practice different cultures and norms. Moreover, speeding is among the most common risky behaviours studied by scholars regardless of nation, whereas violation over the use of mobile phones while driving has been commonly studied among young drivers and university students. In general, the subjective norm indeed plays an important role in avoiding drivers from committing risky driving behaviour. Drivers with high level of sensitivity and concern toward their significant others will tend to abide the traffic rules and regulations to avoid any unwanted incidents that cause disapproval from their significant others. Thus, pressure from the significant others could act as a deterrent towards risky driving.

**Table 1:** Outcome of the relationship between subjective norm and risky driving behaviour

***No***	***Study***	***Country***	***Sample size***	***Behavioural outcome***	***Results***
1.	Åberg and Wallén Warner (24)	Borlänge, Sweden	250 drivers	Self-reported speeding	r= 0.45, *P* < 0.01
2.	Ambak et al (29)	Selangor, Malaysia	300 motorcyclists	Helmet usage	r=0.403, *P* < 0.01
3.	Castanier et al (8)	France	280 drivers	a) Drink-driving b) Excessive speeding c) Following a car too closely d) Using a phone while driving e) Disobey road signs	a) r=0.31, *P* < 0.001 b) r=0.29, *P* < 0.01 c) r=0.19, *P* < 0.01 d) r=0.37, *P* < 0.001 e) r=0.26, *P* < 0.001
4.	Chen and Chen (27)	Taiwan	350 motorcyclists	Speeding behaviour	r= -.0.30 (Not significant)
5.	Cristea and Gheorghiu (13)	France	224 cyclists	a) Intention to run the red light b) Intention to suddenly turn left	a) r= 0.49, *P* < 0.00 b)r= 0.09, *P* < 0.00
6.	Desrichard et al (14)	Grenoble, France	1,654 adolescents	Intention to violate driving rules	r= 0.19, *P* < 0.001
7.	Elliott et al (19)	UK	150 drivers	Self-reported speeding behaviour	Standardized beta weight, β = 0.23, *P* < 0.001
8.	Elliott and Thomson (20)	England, UK	1403 traffic offenders	Subsequent speeding behaviour	r= 0.34, *P* < 0.02
9.	Forward (25)	Sweden	275 drivers	a) Intention to speed b) Intention to dangerous overtake	a) r=0.52, *P* < 0.01 b) r=0.33, *P* < 0.01
10.	Gauld et al (12)	Australia	171 young drivers	Use of mobile phone while driving	r= 0.32, *P* < 0.001
11.	Moan (6)	Norway	1025 random residents	Intention not to ride with an intoxicated driver	r= 0.28, *P* < 0.001
12.	Moan and Rise (7)	Norway	1025 random residents	Intention not to drink and drive	r= 0.14, p < 0.001
13.	Nemme and White (10)	Australia	169 young drivers	a) Sending texts while driving b) Reading texts while driving	a) r= 0.24, *P* < 0.01 b) r= 0.29, *P* < 0.01
14.	Özkan et al (31)	Turkey	451 motorcyclists	Performance of stunt behaviour	Path coefficient = 0.11, *P* < 0.05
15.	Poulter et al (21)	UK	232 truck drivers	Compliance toward traffic law and regulation	Beta weight β = 0.306, *P* < 0.01
16.	Prat et al (30)	Spain	1082 university students	Texting while driving	r= 0.189, *P* < 0.01
17.	Rivis et al (22)	UK	200 male drivers	Drink and drive	r= - 0.34*, P* < 0.001
18.	Rowe et al (23)	Yorkshire, UK	294 college students	a) Driving over the speed limit b) Driving over the legal alcohol limit c) Driving while talking on a hand-held mobile phone d) Driving while feeling very tired	Beta weight, a) β = 0.19, *P* < 0.01 b) β = - 0.08 c) β = 0.19, *P* < 0.05 d) β = 0.14
19.	Tseng et al (28)	Taiwan	544 offenders	Offender driving behaviour	r= 0.03 (Not significant)
20.	Warner and Åberg (26)	Sweden	112 test drive participants	Self-reported speeding	Path coefficient = 0.23, *P* < 0.05

### Relationship and Summary between Group Norm and Risky Driving Behaviour

All three included studies were found to be significant. Two studies from Scotland and France reported that the group norm plays a role in influencing the motorcyclists’ speeding behavior (r= 0.65, *P*<0.01; r= 0.65, *P*<0.01), whereas another study from Australia reported that the young drivers’ behavior of sending and reading texts while driving has significantly put them in danger due to driving distraction (r= 0.38, *P*<0.001; r= 0.18, *P*<0.05) (10, 15). From the studies, the role played by friends is very important because drivers/motorcyclists tend to imitate their friends’ behaviour. For example, the tendency for touring riders to speed was less compared to the sport motorcyclists because the tourists would focus more on enjoying the view and scenery, whereas the sport riders were more likely to enjoy fast and adventure riding (15). Moreover, young drivers can be easily influenced by their friends and behave in accordance with their friends’ behaviour. For example, drivers tend to violate the traffic rules such as running over the red light when his/her friend asks them to do so. On the contrary, driving with friends with a good driving attitude would influence the driver to be in conformity with their friends’ good driving attitude. Table 2 summarized the outcomes of the relationship between group norm and risky driving behaviour.

**Table 2:** Outcome of the relationship between group norm and risky driving behavior

***No***	***Study***	***Country***	***Sample size***	***Behavioural outcome***	***Results***
1.	Elliott (33)	Scotland	110 motorcyclists	Intention to speed	r= 0.65, *P* < 0.01
2.	Eyssartier et al (15)	France	256 sport and touring riders	Intention to exceed the speed limit	r= 0.65, *P* < 0.01
3.	Nemme and White (10)	Australia	169 young drivers	a) Sending texts while driving b) Reading texts while driving	a) r= 0.38, *P* < 0.001 b) r= 0.18, *P* < 0.05

### Summary on the Relationship between Moral Norm and Risky Driving Behavior

Table 3 summarized the outcomes of the relationship between moral norm and risky driving behaviour. Overall, most of the studies were undertaken in Western and European countries such as Sweden, Australia, Norway and the UK except for one study conducted in Iran.

**Table 3:** Outcome on the relationship between moral norm and risky driving behavior

***No***	***Study***	***Country***	***Sample size***	***Behavioural outcome***	***Results***
1.	Åberg and Wallén Warner(24)	Borlänge, Sweden	250 drivers	Self-reported speeding	a) r= - 0.39, *P* < 0.01
2.	Elliott and Thomson (20)	England, UK	1403 traffic offenders	Subsequent speeding behaviour	r= - 0.48, *P* < 0.02
3.	Gauld et al (12)	Australia	171 young drivers	Use of mobile phone while driving	r= - 0.52, *P* < 0.001
4.	Moan (6)	Norway	1025 random residents	Intention not to ride with an intoxicated driver	r= 0.33, *P* < 0.001
5.	Moan and Rise (7)	Norway	1025 random residents	Intention not to drink and drive	r= 0.16, *P* < 0.001
6.	Nemme and White (10)	Australia	169 young drivers	a) Sending texts while driving b) Reading texts while driving	a) r= - 0.42, *P* < 0.001 b) r= - 0.42, *P* < 0.001
7.	Tabibi and Pfeffer (11)	Iran	699 drivers	Intention to comply with traffic rules and regulation	r= 0.44, *P* < 0.001

Speeding and violation over the use of mobile phone were among the common risky behaviours studied by the scholars. Moral norm plays a key role in restraining drivers from committing risky driving behaviour. The drivers with low moral norm were reluctant to abide by the traffic rules and regulation. Unlike the other norms (subjective norm, group norm, injunctive norm and descriptive norms) that highlighted the role of significant others/third parties, moral norm was something decided by the drivers themselves without any outside influence. In another word, drivers are accountable for their own behavior as they commit such mistake on their own willingness. Drivers with low sense of obligation toward the law tend to neglect the traffic law and eventually commit various traffic offences without any sense of guilt.

### Summary of the Relationship between Injunctive Norm and Risky Driving Behaviour

Table 4 summarized the outcomes of the relationship between injunctive norm and risky driving behaviour. Majority of the studies (n=3) were undertaken in France using the sample of young drivers with only one study conducted in the US, Uganda and Iran. Four out of six papers investigated the speeding issue, whereas only two studies discussed the compliance towards the traffic rules and regulation. From the study, the role played by the significant others (family and friends) is crucial in determining the future driving behavior of the driver. For instance, when drivers break the speed limit rule, the reaction of the significant others whether they approved such behavior or not is really important as this will influence the drivers’ future behavior whether to speed again or not. For example, the study by Cestac et al (17) specified that the effects of injunctive norm (whether to approve or not the violation behaviour) played by the parents were not enough to avoid the drivers from violating the speed limit; instead, the parents need to behave accordingly first.

**Table 4:** Outcome on the relationship between injunctive norm and risky driving behavior

***No***	***Study***	***Country***	***Sample size***	***Behavioural outcome***	***Results***
1.	Cestac et al (16)	France	3002 young drivers	Intention to speed	r= 0.22, *P* < 0.01
2.	Cestac et al (17)	France	2428 young drivers	Intention to speed:- a) Injunctive norm (mother) b) Injunctive norm (father) c) Injunctive norm (male friends) d) Injunctive norm (female friends)	a) r= 0.13, *P* < 0.01 b) r= 0.16, *P* < 0.01 c) r= 0.19, *P* < 0.01 d) r= 0.24, *P* < 0.01
3.	Coogan et al (9)	US	990 residents	a) Speeding behaviour b) Aberrant driving	a) r=0.37, *P* < 0.01 b) r=0.32, *P* < 0.01
4.	Delhomme et al (18)	France	1192 young drivers	Intention to speed	r=0.30, *P* < 0.05
5.	Mawanga and Ntayi (32)	Kampala, Uganda	370 drivers	Compliance toward traffic rules	r=0.349, *P* < 0.01
6.	Tabibi and Pfeffer (11)	Iran	699 drivers	Intention to comply with traffic rules and regulation	r= 0.32, *P* < 0.001

### Summary on the Relationship between Descriptive Norm and Risky Driving Behaviour

Table 5 summarized the outcomes of the relationship between descriptive norm and risky driving behaviour. Overall, most of the studies were undertaken in Western and European countries such as Sweden, France, Norway, the US and the UK. Speeding, drink-drive and compliance to traffic rules and regulation were among the most risky driving behaviours studied by the researchers. Besides, these studies have employed various types of research sample such as young drivers, traffic offenders as well as random residents. From the findings, the practice of the descriptive norm through notifying the drivers to drive safely and abide with traffic rule and regulation has been proven ineffective in overcoming traffic offences. Advising the drivers not to break the law alone is not enough when the significant others (family and friends) themselves also break the law. The drivers tend to think that if their significant others can do so, it is sensible to act similarly. For example, the common message of “please drive as I said, but do not drive as I am” made by parents is irrelevant in avoiding the young drivers from speeding (17). Children learn through the parents’ behaviour; therefore, it is sensible for the parents to set a good example.

**Table 5:** Outcome on the relationship between descriptive norm and risky driving behavior

***No***	***Study***	***Country***	***Sample size***	***Behavioural outcome***	***Results***
1.	Cestac et al (16)	France	3002 young drivers	Intention to speed	r= 0.30, *P* < 0.01
2.	Cestac et al (17)	France	2428 young drivers	Intention to speed:- a) Injunctive norm (mother) b) Injunctive norm (father) c) Injunctive norm (male friends) d) Injunctive norm (female friends)	a) r= 0.19, *P* < 0.01 b) r= 0.21, *P* < 0.01 c) r= 0.30, *P* < 0.01 d) r= 0.26, *P* < 0.01
3.	Coogan et al (9)	US	990 residents	a) Speeding behaviour b) Aberrant driving	a) r=0.44, *P* < 0.01 b) r=0.42, *P* < 0.01
4.	Elliott and Thomson (20)	England, UK	1403 traffic offenders	Subsequent speeding behaviour	r= 0.37, *P* < 0.02
5.	Forward (25)	Sweden	275 drivers	a) Intention to speed b) Intention to dangerous overtake	a) r=0.49, *P* < 0.01 b) r=0.51, *P* < 0.01
6.	Mawanga and Ntayi (32)	Kampala	370 drivers	Compliance toward traffic rules	r=0.545, *P* < 0.01
7.	Moan (6)	Norway	1025 drivers	Intention not to ride with an intoxicated driver	r= 0.19, *P* < 0.001
8.	Moan and Rise (7)	Norway	1025 drivers	Intention not to drink and drive	r= - 0.18, *P* < 0.001
9.	Tabibi and Pfeffer (11)	Iran	699 drivers	Intention to comply with traffic rules and regulation	r= 0.42, *P* < 0.001

## Conclusion

Norms indeed play a role in influencing drivers’ risky driving behaviour. This is consistent with the previous studies that suggested norm as an important variable that needs to be put into consideration as one of the measures in overcoming the road accident problems. Nevertheless, the relationship between subjective norm and risky driving behavior reported mixed findings with two studies conducted in Taiwan, reported insignificant. Research setting plays a role in influencing the research outcome as culture and norm practiced in Taiwan are different compared to those in other countries. Moreover, most of the studies were conducted in European and Western countries like France, the UK and Sweden with only few studies conducted in Asian and Middle East settings. This suggests that more studies from various research settings are needed to enrich the body of knowledge. Subsequently, young drivers and university students were those who regularly commit risky driving behavior especially, through the use of mobile phone and speeding. Finally, this review study can help in revealing the impact of norms toward driving behaviour, which can further aid the related bodies to outline appropriate educational awareness. Much attention is required to educate the society in practicing and cultivating good driving norms. Apart from that, parents also play a vital role in influencing their children driving behaviour. Rather than just advising the children to drive safely, they also need to abide by the traffic rules and regulations to set a good example for their children.

## Ethical considerations

Ethical issues (Including plagiarism, informed consent, misconduct, data fabrication and/or falsification, double publication and/or submission, redundancy, etc.) have been completely observed by the authors.
